# Polyphenols and Cardiovascular Health: Emerging Relevance for Blueberries, Grapes, and Red-Fleshed Table Grapes

**DOI:** 10.3390/nu18121968

**Published:** 2026-06-18

**Authors:** Emma J. Derbyshire, José A. Abellán-Alemán, Nisa Aslam

**Affiliations:** 1 Nutritional Insight, Epsom KT17 2AA, UK; 2 Cátedra de Riesgo Cardiovascular, Universidad Católica de Murcia, 30107 Murcia, Spain; jabellan@ucam.edu; 3 West Hertfordshire Teaching Hospitals NHS Trust, Watford WD18 0HB, UK; nisa.aslam@nhs.net

**Keywords:** anthocyanins, berries, bioactives, blood pressure, cardiovascular health, endothelial function, grapes, polyphenols, red-fleshed table grapes, resveratrol

## Abstract

**Background**/**Objectives**: This review aimed to provide an updated synthesis of the evidence on the effects(s) of grapes, blueberries, and their constituent bioactives on cardiovascular health. Cardiovascular disease remains one of the most prevalent non-communicable diseases globally. **Methods**: A systematic literature search was conducted using PubMed, Science Direct, and Semantic Scholar. Eligible publications were restricted to studies published since 2015 focusing on grapes, blueberries, and related bioactives. A total of 37 studies were included (17 meta-analyses/systematic reviews and 20 randomised controlled trials). Compositional data on polyphenols, anthocyanins, and stilbenes (including resveratrol) from a new hybrid variety of red-fleshed table grape were also discussed in context. **Results**: The evidence indicates that grape- and blueberry-derived bioactives, particularly polyphenols and resveratrol, produce modest but consistent improvements in cardiovascular risk markers, particularly endothelial function. Effects were more pronounced in higher-quality trials and in metabolically at-risk populations. Certain varieties, including red-fleshed table grapes (red berry grapes), may represent effective dietary sources of these bioactives. **Conclusions**: Cardiovascular disease remains a common public health challenge. Increasing attention is being given to dietary and lifestyle strategies for its prevention and management. Within this context, and alongside existing recommendations to increase fruit and vegetable intake, there is scope for more specific guidance emphasising the consumption of dark-pigmented grapes, berries, and red-fleshed table grapes abundant in bioactives such as polyphenols.

## 1. Introduction

Cardiovascular (CV) disease is a collective term that encompasses disorders of the heart and blood vessels, including cerebral, coronary, peripheral, and other vascular diseases affecting the transport of blood and circulation [1]. CV disease remains a leading cause of premature mortality and death in Europe, with more than 60 million potential years of life lost due to its onset [2]. Across the European Union, the prevalence of CV disease varies by age, gender, and population subgroup, and alongside population ageing, it contributes to rising multimorbidity and increasing pressure on healthcare systems [3]. CV disease imposes an annual cost of €282 billion across the European Union, which is equivalent to 11% of total health expenditure—including €155 billion (55%) for health and long-term care, €79 billion (28%) for informal care, and €48 billion (17%) in productivity losses [4].

In the UK, the overall burden of CV diseases remained high from 2000 to 2019, with future prevention strategies especially needed for younger age groups and socioeconomically deprived populations [5]. In terms of gender differences, although age-standardised CV disease rates are higher in men, a greater absolute number of women die from CV disease, reflecting differences in population age structure and survival [2]. CV disease is traditionally treated through medications, although the roles of integrative medicine and prevention from a nutrition perspective are expanding [6,7,8]. In particular, there is increasing interest in the potentially promising role(s) of dietary bioactives in the prevention and treatment of CV disease [9].

From a bioactive compound perspective, grapes and blueberries share similar phytochemical profiles that may confer CV health benefits [10]. In botanical terms, berries are defined as fruits that develop from the ovary of a single flower and contain seeds; under this classification, the category includes the following: blueberries, cranberries, lingonberries, Ribes species, and grapes [11]. The biological activities of grapes and blueberries have mainly been attributed to their diverse array of flavonoids (anthocyanins, flavanols, and flavonols), phenolic acids, tannins, and lignans [11,12,13,14]. There is growing evidence that polyphenols in plant foods (e.g., grapes and blueberries) could protect against atherosclerosis, dyslipidaemia, endothelial dysfunction, hypertension, and inflammation—all predominant contributors to CV disease risk [15]. Diet is increasingly being recognised as a modifiable risk factor for CV disease, and it has previously been proposed that grapes and other berries could be eaten as part of a heart-healthy diet [10]. This review updates the evidence since the 2015 grapes/berries and CV health publication [10]. It collates findings on grapes, blueberries, and related bioactives, integrating mechanistic insights and translating these data to red-fleshed table grapes, with an emphasis on their distinct polyphenol profiles, cardioprotective potential, and promise as a functional dietary strategy.

## 2. Materials and Methods

### 2.1. Search Strategy

A comprehensive search of PubMed for meta-analysis (MA), systematic review (SR), and randomised controlled trial (RCT) publications was undertaken for the last 11 years (1 January 2015–30 April 2026), using a combination of MeSH terms and free-text keywords. The search was conducted from 2015, so the present review provided an update on Wightman and Heuberger, which was published in 2015 [10]. A secondary search of Science Direct and Semantic Scholar was also conducted to capture any additional publications, excluding conference abstracts and replica publications.

For this review, the search terms “grapes”, “red fleshed grapes”, “Vaccinium”, or “blueberries/blueberry”, “polyphenols”, “flavonoids”, and “anthocyanins” were used. The outcome terms included “cardiovascular diseases”, “endothelium/endothelial function”, “blood pressure”, “platelet aggregation”, “thrombosis”, “dyslipidemias”, “low-density lipoprotein (LDL) oxidation”, “inflammation”, “platelet aggregation”, “lipid profile”, “oxidative stress”, “flow-mediated dilation”, and “arterial stiffness”. A full list of search terms is included in the Appendix A. From the initial search, titles and abstracts were first reviewed and publications identified as candidates for full-text screening. Publication reference lists were also screened to identify additional publications.

### 2.2. Inclusion and Exclusion Criteria

**Figure 1:**
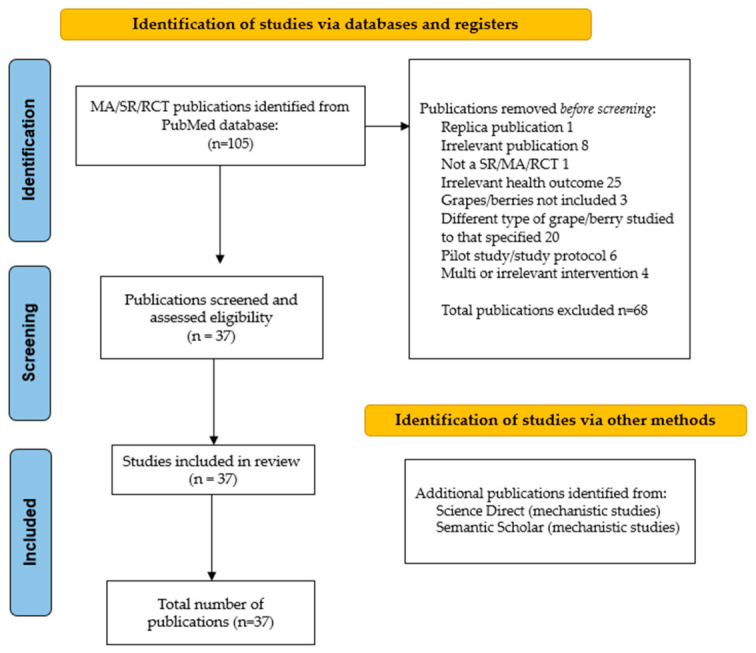
PRISMA 2020 algorithm and flow diagram, which included searches of databases and other sources. Adapted from Page et al. (2021) [16].

The review process followed the Preferred Reporting Items for Systematic Reviews and Meta-Analyses (PRISMA) statement [16]. Figure 1 shows the PRISMA study selection flow diagram of included searches of databases and other sources. Studies were included if they met the following criteria: (1) human studies, (2) published in the English language, (3) study design was a MA, SR, or RCT (filters were applied), (4) included an intervention that involved grapes, red-fleshed table grapes (red berry grapes), or blueberries, (5) focused on key bioactives found in grapes/blueberries, e.g., polyphenols, flavonoids, anthocyanins, and resveratrol, which is a stilbene molecule belonging to the polyphenol family [17], or (6) measured at least one cardiovascular outcome (e.g., blood pressure, endothelial function, low-density lipoprotein (LDL) oxidation, inflammatory biomarkers, or platelet aggregation).

Studies were excluded if they met the following criteria: (1) focused solely on animal models or in vitro experiments (except for relevant mechanistic evidence), (2) assessed mixed polyphenol supplements, (3) were multi-interventions, or (4) lacked clear cardiovascular endpoints. This approach allowed for a targeted synthesis of high-quality clinical evidence on the cardiovascular effects of blueberries, grapes, and related bioactives, emphasising mechanistic outcomes as well as clinically relevant biomarkers. E.J.D. identified key publications for inclusion within the review and J.A.A./N.A. undertook a cross-check.

### 2.3. Quality of Publications

The quality of the included publications was evaluated using validated appraisal tools. The AMSTAR-2 checklist comprises 16 items and was used to evaluate the methodological quality of MAs/SRs [18,19]. Studies were classified as critically low, low, moderate, or high quality in accordance with established AMSTAR-2 guidance [18,19]. For RCTs, the Jadad scale was used, with total scores ranging from 0 to 5 [20]. Scores of 0–2 were considered indicative of low quality, a score of 3 represented moderate quality, and scores of 4–5 were suggestive of high quality.

## 3. Results

### 3.1. Meta-Analysis and Systematic Review Publications

As shown in Table 1, 17 key MA/SR publications have been published since 2015 examining grapes, blueberries, related bioactives, and markers of CV health. Of these, two were rated as being of high quality [21,22]. Sarkhosh-Khorasani et al. (2021) conducted a SR and MA of 17 RCTs (*n* = 668 adults) and found that grape polyphenols (>500 mg/day) significantly reduced the amount of C-reactive protein (CRP, a marker of inflammation) over ≥12 weeks, with effects varying by health status and threshold dose response [21]. Weaver et al. (2021) undertook an SR and MA of 37 human studies, reporting that red wine polyphenols improved vascular function and blood pressure in 84% of studies, with significant reductions in systolic blood pressure observed [22]. Both were rated high quality for appropriately accounting for intervention and population variability [21,22].

Two moderate-quality reviews also reported CV benefits: Lagou et al. (2025) in an SR and MA of 145 RCTs (*n* = 5202) found that flavan-3-ol foods (cocoa, tea, apples, and grape-derived products; ≈586 mg/day) modestly reduced blood pressure and improved endothelial function, suggesting potential CV benefits [23], and an SR and MA of 30 RCTs showed whole grape product consumption to be associated with significant systolic blood pressure reductions [24].

The remaining publications were generally rated low or critically low quality due to methodological limitations upon examining oxidative stress, inflammation, blood pressure, endothelial function, and metabolic markers [25,26,27,28,29,30,31,32,33,34,35,36,37]. Notable findings included grape products reducing total cholesterol, LDL, and triglycerides (Ghaedi et al., 2019; 48 RCTs) [31]; benefits on lipid levels and blood pressure in adults with metabolic syndrome (da Silva et al., 2023) [35]; improved oxidative stress biomarkers with grape seed extract (Foshati et al., 2021; 19 studies) [27]; and associations between resveratrol and improved blood lipids, blood pressure, and inflammatory markers [25,30].

Overall, higher-quality evidence indicates that grape- and blueberry-derived bioactives, particularly polyphenols, flavan-3-ols, and resveratrol, produce modest but consistent improvements in CV risk markers, including blood pressure, vascular/endothelial function, and inflammatory profiles [21,22,23]. Lower-quality reviews broadly align, indicating improvements in oxidative stress and lipid and metabolic markers, but with greater levels of methodological uncertainty.

**Table 1:** MA/SR publications focusing on berry fruits/related bioactives and markers of CV health.

Study (Author, Year, Location)	Study Design	Sample Size	Bioactive Source	Bioactive Compound(s)	Intervention (Dosage/Amount)	Outcomes	Main Cardiovascular Findings	AMSTAR-2 Quality
Shen et al. (2026) [25], Taiwan	SR	40 RCTs, *n* = 2551	Supplementation	Resveratrol	N/A	CV markers	Resveratrol significantly ↓ HOMA-IR, TC, TG, LDL cholesterol, SBP, DBP, hs-CRP, TNF-α and IL-6	CLQ
Lagou et al. (2025) [23], Netherlands	SR and MA of RCTs	109 studies including 145 RCTs, *n* = 5205	Epicatechin, EGCG, cocoa products, tea, grape extract, and apple interventions	Flavan-3-ols	Flavan-3-ol interventions delivering 586 mg	BP and endothelial function	Flavan-3-ol-rich foods ↓ elevated BP and improved endothelial function, supporting their use for CV prevention	MQ
Ashoori et al. (2023) [24], Iran	SR and MA	30 RCTs	Whole grape products	Various—not specified	N/A	BP and vascular function	Grape product consumption: ↓ SBP (−3.17 mmHg; *p* = 0.004) ↑ VCAM-1 (+34.11 ng/mL; *p* = 0.04)	MQ
da Silva et al. (2023) [35], Brazil	SR and MA	27 RCTs	Grape products	Various—not specified	N/A	MetS risk factors	Grape products ↓ total and LDL cholesterol and TG levels, grape supplementation also ↓ SBP and DBP	LQ
Martini et al. (2023) [26], Italy	SR	45 human intervention studies	Blueberries	Polyphenols	N/A	CV function markers, oxidative stress, and inflammation	Blueberries may play a role in the improvement of markers of vascular function postprandially and long-term, especially in subjects with disease conditions or risk factors	CLQ
Foshati et al. (2021) [27]	SR and MA RCTs	SR *n* = 23 studies MA *n* = 19 studies	Grape seed extract	Various—not specified	N/A	Inflammation and oxidative stress	↓ in MDA (−1.04, 95% CI: −1.65, −0.42), ↓ oxidised LDL (−0.44, 95% CI: −0.75, −0.13), ↓ hs-CRP (−0.48 mg/L, 95% CI: −0.94, −0.03), ↑ TOD (0.49, 95% CI: −0.05, 1.04)	LQ
Sarkhosh-Khorasani et al. (2021) [21], Iran	SR and MA	17 RCTs, *n* = 668	Grape, grape seed, grape seed extract, and wine	Polyphenols	Higher doses of grape polyphenols (>500 mg/d) and longer intervention periods (≥12 weeks) had significant effects	CRP levels	↓ CRP (SMD = −0·229; 95% CI −0·41, −0·05; *p* = 0·013)	HQ
Weaver et al. (2021) [22], UK	SR and MA	48 animal and 37 human studies	Red wine	Polyphenols	N/A	Vascular health	Human studies = significant improvements in SBP overall (−2.6 mmHg, 95% CI: [−4.8, −0.4]), with a greater improvement in pure-resveratrol studies alone (−3.7 mmHg, 95% CI: [−7.3, −0.0])	HQ
Haghighatdoost et al. (2020) [29], Iran	SR and MA	8–9 publications included	Grape, grape extract, grape juice, and grape seed extract	Polyphenols	N/A	Inflammatory mediators	No beneficial effects of grape polyphenols on selected inflammatory mediators were observed	CLQ
Koushki et al. (2020) [28], Iran	SR and MA	12 RCTs	Grapes and red wine	Resveratrol	N/A	Oxidative stress	RCTs did not show any benefit of resveratrol supplementation on SOD, CAT, or GPx except for TAC	LQ
Mashhadi et al. (2020) [30], Iran	SR	5 studies *n* = 229 hypertensive and pre-hypertensive patients	Various	Resveratrol	N/A	BP	Resveratrol appears to play an important role in reducing BP and there was an indication of a small ↑ in FMD with berries	CLQ
Ghaedi et al. (2019) [31], Iran	SR and MA	48 RCTs (59 arms)	Grape products	Various—not specified	N/A	Blood lipids	Grape products ↓ the concentration of TC (MD: −6.196 mg dL^−1^, 95% CI: −9.203, −3.189), LDL cholesterol (MD: −4.964 mg dL^−1^, 95% CI: −7.594, −2.334), and TG (MD: −7.641 mg dL^−1^, 95% CI: −12.120, −3.162)	LQ
Garcia-Conesa et al. (2018) [37], Spain	MA	128 studies	Berries and red grapes/wine	Anthocyanins	N/A	Cardiometabolic biomarkers	BP was significantly reduced by the two main sources of anthocyanins, berries, and red grapes/wine	CLQ
Marx et al. (2017) [32], Australia	SR and MA	12 studies	Polyphenol-rich interventions including grape, soy, cocoa, pomegranate, and turmeric	Polyphenols	N/A	CV risk factors in haemodialysis, BP, TG, and vascular function	Polyphenol-rich interventions significantly improved DBP (MD: −5.62 mmHg, 95% CI: −8.47, −2.78; I^2^ = 2%; *p* = 0.0001) and TG levels (MD: −26.52 mg/dL, 95% CI: −47.22, −5.83; I^2^ = 57%; *p* = 0.01)	LQ
Woerdeman et al. (2017) [33], Netherlands	SR	39 studies	Grapes	Polyphenols	N/A	MetS	One out of two high-quality studies noted improvements in insulin sensitivity. Seven out of twenty-two studies had a significant ↓ in BP, but only one was of high quality	LQ
Zhu et al. (2017) [34], China	SR and MA RCTs	6 RCTs *n* = 204	Blueberries	Polyphenols/anthocyanins	N/A	BP (SBP, DBP)	There was no significant effect of blueberry supplementation on changes in BP relative to the baseline	CLQ
Li et al. (2015) [36], China	MA of RCTs	10 studies	Grapes	Polyphenols	N/A	BP	Daily grape polyphenol intake significantly reduced systolic blood pressure by 1.48 mmHg when compared to control subjects (*p* = 0.03)	CLQ

Key: BP, blood pressure; CAT, catalase; CI, confidence interval; CRP, C-reactive protein; CV, cardiovascular; DBP, diastolic blood pressure; EGCG, epigallocatechin-gallate; FMD, flow-mediated dilation; GPx, glutathione peroxidase; HOMA-IR, homeostatic model assessment of insulin resistance; hs-CRP, high-sensitivity C-reactive protein; IL-6, interleukin-6; LDL, low-density lipoprotein; MA, meta-analysis; MDA, malondialdehyde; MD, mean difference; MetS, metabolic syndrome; N/A, not applicable; RCT, randomised controlled trial; SBP, systolic blood pressure; SMD, standardised mean difference; SOD, superoxide dismutase; SR, systematic review; TAC, total antioxidant capacity; TC, total cholesterol; TG, triglycerides; TNF-α, tumour necrosis factor alpha; TOD, total oxidative damage, VCAM-1, vascular cell adhesion molecule-1, ↑ increased and ↓ decreased. AMSTAR-2 Ratings: CLQ, critically low quality; LQ, low quality; MQ, moderate quality; and HQ, high quality.

### 3.2. Randomised Controlled Trials

Twenty key RCTs were identified examining grapes, blueberries, related bioactives, and CV health markers (Table 2). Trials were generally small to moderate in scale (*n* ≈ 20–115) and short-term, with occasional acute postprandial designs [38,39]. Interventions clustered around berry-derived polyphenols—principally blueberries (including freeze-dried), grapes, grape seed extract/pomace/powder, and resveratrol, with outcomes spanning blood pressure [40,41,42,43,44,45], lipid profiles [46,47,48,49], endothelial function (e.g., flow-mediated dilation and reactive hyperaemia index) [42,44,50,51,52], oxidative stress (malondialdehyde, TBARS, and total antioxidant capacity) [49,53], and inflammatory markers (TNF-α and IL-6) [38,53]. Seventeen studies were rated high or medium quality [38,39,41,42,43,44,45,46,47,48,50,51,52,53,54,55,56] and three of lower quality [40,49,57], with stronger methodological designs generally associated with more consistent vascular and lipid-related effects [41,42,44,45,46,56].

Nine key studies focused on whole grapes, grape pomace, grape seed extracts, or red grape cell powder [39,40,41,44,47,48,49,56,57], generally demonstrating favourable effects on CV risk markers. Higher-quality RCTs reported improvements in lipid profiles [47,48], flow-mediated dilation, lipid peroxidation and reduced diastolic blood pressure [44], and paraoxonase activity—indicating an enhanced lipoprotein-associated antioxidant capacity [48]. Lipid improvements were frequently accompanied by reductions in oxidative stress (e.g., TBARS) [49] and markers of endothelial dysfunction (endothelin-1 and sICAM-1) [56], with effects being more pronounced in hypercholesterolaemic or metabolically at-risk populations [41,47,48,49,57]. For example, whole red grape consumption (500 g/day over 8 weeks) was associated with reductions in total and LDL cholesterol levels in adults with hypercholesterolaemia at baseline [49]. Although effect sizes vary across formulations and study designs, the overall pattern suggests a coherent cardiometabolic benefit.

Blueberry and resveratrol interventions primarily demonstrated vascular and endothelial benefits, including improved flow-mediated dilation and nitric oxide production and reduced inflammatory markers and blood pressure—with more limited or inconsistent effects on lipid endpoints [42,45,46,51,53,54,55]. Curtis et al. (2019) conducted one of the strongest studies, with one cup of blueberries (150 g) daily over six months significantly improving endothelial function and arterial stiffness, contributing to effect sizes predictive of 12–15% reductions in CVD risk [52].

Taken together, RCT evidence suggests that berries (grapes and blueberries) and their derived polyphenols confer modest but consistent cardiometabolic effects, most reliably observed in improvements in vascular function [41,42,44,50], oxidative stress [48,49], and certain lipid parameters [46,47,48,49]. Effects are generally more pronounced in higher-quality trials and in metabolically at-risk populations, although findings remain constrained by small sample sizes, short durations, and reliance on surrogate endpoints rather than clinical outcomes.

## 4. Bioactives—Potential Mechanisms of Action

Several fruits have been identified as “nutritional fruits” with scope to protect against CV diseases, with grapes being well explored and enlisted amongst these [58]. Grapes and berries provide an array of polyphenolic bioactive compounds, including anthocyanins, flavanols, flavonols, and resveratrol, which exhibit multiple biological activities, including cardioprotective, antioxidant, and anti-inflammatory effects [12,59].

Polyphenols in particular appear to confer promising CV benefits by enhancing endothelial function through increased nitric oxide bioavailability, reducing LDL oxidation, and modulating cardiomyocyte signalling by suppressing inflammatory marker expression [60]. Experimental evidence also indicates that grape polyphenols may attenuate CV conditions such as atherosclerosis by reducing LDL oxidation and platelet aggregation, suppressing inflammation, improving endothelial function and blood pressure, and activating anti-senescence pathways (e.g., Sirtuin 1) [61]. Emerging evidence further indicates that polyphenols, particularly anthocyanins, are metabolised by gut microbiota into bioactive metabolites that could further influence CV function [62].

Other research suggests that resveratrol modulates cardiac remodelling through its effects on intracellular signalling pathways, while exerting anti-inflammatory and antioxidant actions and enhancing mitochondrial biogenesis and the degradation of damaged mitochondria [63]. Within heart tissues, resveratrol may also prevent oxidative stress by regulating proteins that induce oxidation [60]. Subsequently, it is thought that dietary polyphenols could have an important preventative role in helping to offset heart diseases such as hypertension, ischemic heart disease, strokes, and heart failure [60].

## 5. Polyphenol Composition of Red-Fleshed Table Grapes

Polyphenols are widely distributed in an array of plant foods but are predominantly found in fruits such as grapes and other berries, cocoa, vegetables, tea, coffee, and other plant foods [64]. Polyphenols, also referred to as phenolic compounds, are a diverse group of secondary bioactive compounds [65]. These include flavonoids such as anthocyanins, flavanones, flavonols, and flavan-3-ols, alongside non-flavonoids, such as stilbenes [65].

Grapes in particular are renowned for the array of bioactive constituents in their fruits, seeds, stems, skins, and pomaces [66]. They are important dietary sources of polyphenols, including proanthocyanins, anthocyanins, and resveratrol, with grape seeds, skins, and pulp typically providing the highest to lowest total phenolic content, respectively [67]. New red-fleshed table grape (red berry grape) varieties have been developed using conventional hybridisation techniques, incorporating wine grape cultivars to introduce the genetics responsible for the teinturier (red-fleshed) pulp into white-fleshed table grapes, thereby enabling polyphenol accumulation within the pulp [68]. Where the female parent was seedless, embryo rescue was used, with embryos cultured in vitro upon maturity, then transplanted to a greenhouse, and ultimately established in the field to generate new varieties [68].

### 5.1. Total Phenolic Content

**Figure 2:**
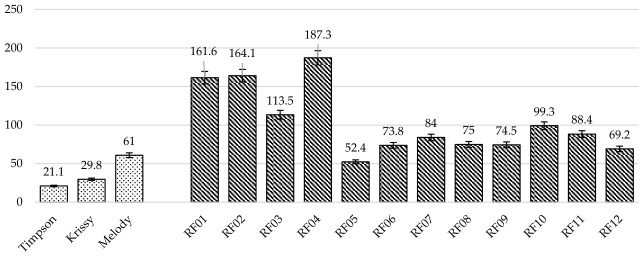
Total phenolic compounds in red-fleshed grapes (mg/100g FW). Key: RF, red-fleshed. Bars represent the standard deviation.

The total phenolic content of red-fleshed table grapes has been previously determined using the validated Folin–Ciocalteu method [68,69]. As shown in Figure 2, all red-fleshed table grape hybrids had a significant increase in the total content of phenolic compounds compared to standard grape varieties (Timpson^TM^ (white grape), Krissy^TM^ (red grape), and Melody^TM^ (black skin colourless pulp)) [68]. The new red-fleshed hybrid varieties had higher phenolic profiles (52.4–187.3 mg/100 g FW), surpassing Melody^TM^ (61 mg/100 g FW) in most instances [68].

Several publications have determined the total phenolic content across different fruits [68,70,71,72,73,74,75]. Overall, tropical fruits and blueberries appear to be particularly high in phenolics [71,73,75,76], followed by fruits such as apples and strawberries [74,77], whilst pears, melon-type fruits, and some apples are generally lower-phenolic-compound fruits [72,76,78]. Red-fleshed table grapes (red berry grapes) tend to have higher total phenolic contents than standard grapes, potentially positioning them as a moderate- to higher-phenolic-compound fruit [68]. However, comparisons of total phenolic contents between fruits should be interpreted with caution, due to variability in analytical methodologies, extraction protocols, reporting units, and seasonal variation.

### 5.2. Total Anthocyanins

**Figure 3:**
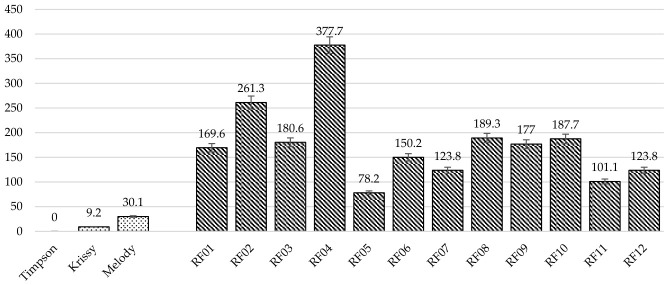
Total anthocyanin compounds in red-fleshed grapes (mg/100 g FW). Key: RF, red-fleshed. Bars represent the standard deviation.

It is well recognised that berries with red, blue, or purple pigments are particularly important providers of anthocyanins [79,80,81]. The total anthocyanin concentration within red-fleshed table grapes has been determined using absorbance methods (Figure 3) [68,82]. Among hybrids, anthocyanin levels ranged from 78.2 to 377.7 mg/100 g FW [68]. RF04 had the highest anthocyanin concentration (337.7 mg/100 g FW), which was nearly ten times higher than standard Melody^TM^ (30.1 mg/100 g FW) grape varieties [68]. Apples, peaches, and plums tend to have lower anthocyanin concentrations compared to berry fruits, particularly black/darker varieties [81]. However, it should be considered that anthocyanin quantification is highly method-dependent, and there is a need for greater uniformity of methods to aid cross-study comparisons [83].

### 5.3. Stilbene/Resveratrol Profiles

Resveratrol is a stilbene molecule belonging to the polyphenol family [17] and is produced as part of the defence mechanism against insect herbivores and microbes [84]. It is most abundant in the skins of dark grapes [85] but also found in other parts of the plant such as the leaves, roots, and shoots, as well as in grape-derived products like juice, raisins, powders, and grape pomace [86]. It is found in a range of foods including grapes, other berries, and peanuts, with grapes and their derivative products being some of the most abundant natural sources [87]. Resveratrol exists naturally in both trans- and cis- isomeric form, with each proposed to have different biological effects [88].

Levels of stilbenes in red-fleshed table grapes have been measured using High-Performance Liquid Chromatography with Diode Array Detection [68]. The total stilbene levels in the five samples analysed ranged from 781.3 to 3913.4 µg/100 g FW, with trans-resveratrol, cis-piceide, and viniferine being some of the most predominant compounds, while cis-resveratrol was absent in some hybrids [68]. Levels of trans-resveratrol ranged from 243.9 to 2480.2 µg/100 g FW [68]. Research has previously demonstrated that trans-resveratrol appears to be the predominant stilbene in grapes, with cis-resveratrol typically being absent [89,90,91] and factors such as grape variety/genetic background, external stimuli, and stage of ripening affecting levels [84,91,92].

Other preliminary research has measured the trans-resveratrol levels in different fruits, which were found to range from 0.2 µg/g (tomato and strawberry) to 3 µg/g in dates [88]. This shows that red-fleshed table grapes (red berry grapes) can have similar or much higher levels of trans-resveratrol than such fruits (red-fleshed grape conversion: 2.4–24.8 µg/g) [68,88]. Some acute studies suggest that trans-resveratrol supplementation could benefit endothelial function and markers of metabolic syndrome, e.g., insulin sensitivity and glucose homeostasis [93,94], but these were acute supplementation/pilot trials conducted with subjects with elevated metabolic parameters at baseline.

## 6. Whole-Food Dietary Patterns and Heart Health

Whilst fruits and vegetables are integrated into many public health guidelines, fruits are of particular interest as they tend to be higher in phenolic compounds than vegetables [95]. Amongst European regions, Norway specifically advises that “*Fruit, berries or vegetables should be eaten with every meal and can also be enjoyed as a snack. It is recommended that you eat at least five and preferably eight servings a day. Vary between different types of fruits, berries, and vegetables*” [96]. One serving is defined as 100 g, which constitutes about a handful of vegetables, fruit, or berries [96]. Within the UK, the Eatwell guide advises the consumption of at least five portions of a variety of fruit and vegetables daily, with a portion defined as 80 g [97]. Health promotion campaigns are also increasingly advocating for the ‘eat a rainbow’ concept and the value of ‘colour density’, given that colour-associated fruit and vegetable variety can provide an array of bioactive pigments that may confer health benefits [98,99].

Diet and nutrition can also play a central role in CV disease prevention [7,8,100], and interest in plant-based diets for CV health is growing [101]. For example, the European Association of Preventive Cardiology and the Association of Cardiovascular Nursing & Allied Professions of the European Society of Cardiology state that plant-based dietary patterns rich in minimally processed foods, vegetables, and fruits may reduce CV disease risk [8]. Fruit polyphenols have been associated with certain health benefits, e.g., reduced risk of CV disease [102]. Some organisations such as the American Heart Association (AHA) provide dietary guidance that includes following certain dietary patterns, such as plenty and a variety of fruits and vegetables to promote cardiometabolic health [103]. Heart Research UK has undertaken heart-healthy cooking programmes to raise awareness in deprived regions of Scotland, UK about the value of cooking and nutrition [104]. The British Heart Foundation is aligned with the UK Eatwell Guide and advises eating a variety of fruit and vegetables, with a portion constituting 80 g [105]. Heart UK also provides similar advice, with more of a cholesterol focus [104].

## 7. Discussion

Overall, CV disease remains a leading cause of death in Europe and globally, including disproportionate effects of CV outcomes on women and certain ethnic and racial groups [2,106,107]. Whilst it is well recognised that certain dietary patterns including regular fruit and vegetable consumption may reduce CV disease risk [7,103,108,109], the specific roles of dark fruits, e.g., red grapes and blueberries and their polyphenol bioactive profiles often remain overlooked.

In 2015, it was suggested that grapes and other berries could be incorporated into heart-healthy dietary recommendations [10]. Similarly, Sabra et al. (2021) also concluded that grapes and their constituent bioactives could be regarded as a potential functional food in cases of hypertension [110]. Now, over a decade on from 2015, the body of evidence has continued to accrue. This publication shows that at least a further 17 key MA/SR publications and 20 RCTs have been published in the last decade. The overarching totality of evidence indicates that grape- and berry-derived bioactives produce small but reproducible improvements in CV risk markers. Effects appear to be particularly consistent across outcomes such as blood pressure, endothelial/vascular function, and inflammation, although it should be considered that effect sizes are modest. Baseline population characteristics should also be considered, as the cardiometabolic benefits of berry-derived bioactives appear more pronounced in individuals with elevated baseline risk, e.g., overweight adults [47] and those with prehypertension [41], metabolic syndrome risk factors [57], hypercholesterolaemia [49], or hyperlipidaemia [48], suggesting how potential effects could be modified by underlying metabolic status. Of the outcomes studied, the most consistent improvement appears to be in endothelial/vascular function, as observed in higher quality RCTs [39,41,42,50,51]. This appears to align with underpinning mechanisms, including polyphenol bioactivity and nitric oxide modulation [45,60]. Similarly, research conducted with red-fleshed apples has also documented improvements in endothelial function and inflammation [111].

Based on the science over the last 11 years, there appears to be potential scope to include dark fruits such as blueberries, red grapes, and red berry grapes within heart-health dietary messages [10,68]. Increasingly, whole plant extracts and/or bioactive compounds are showing preventative and/or therapeutic effects for certain diseases due to an array of underpinning biological effects [112]. Alongside growing awareness of nutrient density, we are now also seeing greater interest in ‘colour density’, with an ‘eat by colour’ approach proposed by some scientists, although there may be some ambiguity in associating certain fruits with specific health outcomes [98,99]. There is also much wider interest in polyphenols from a health perspective, with indices such as the (poly)phenol-rich diet score now being developed [113]. Similarly, an earlier phytochemical index used to determine the percentage of dietary calories from foods rich in phytochemicals was compiled and could be applied to foods such as grapes/other berries [114,115].

Finally, it is also important to consider research limitations and future directions. Firstly, most MA/SR and RCT publications focused on CV biomarkers, such as blood pressure or endothelial function rather than CV events, e.g., myocardial infarction or stroke. This is important to consider as there could be gaps between biomarker improvements and real-world clinical benefits [116,117]. Secondly, larger and longer RCTs are also needed, specifically focusing on using new red-fleshed berry varieties. This would include more studies using whole grapes/berries rather than extracts or freeze-dried concentrates. It has also been proposed that sex-dependent blood pressure responses to polyphenols may exist; therefore, ongoing research is warranted [118]. Thirdly, several publications focused on resveratrol supplementation [25,53], while others examined red wine polyphenols [22,37]. Although these studies provide valuable physiological and mechanistic insights into the potential cardiometabolic effects of polyphenols, several limitations should be acknowledged. The levels of bioactives would have been highly concentrated—beyond that typically found in fresh produce. Furthermore, because red wine contains alcohol and polyphenols it has an unusual matrix, which may affect polyphenol bioavailability and physiological effects [119]. Regarding outcomes such as vascular function, many patients were receiving medications, which could have introduced additional variability and confounded the results [22]. Consequently, caution is warranted when extrapolating these findings to real-world populations consuming foods rich in polyphenols as part of a habitual diet. Finally, the AMSTAR and Jadad tools were used to assess the quality of evidence [18,19,20]. For both, future modifications are needed to further improve their reliability, usability, and validity; they are a guide rather than the gold standard [120,121,122].

## 8. Conclusions

Overall, the available evidence indicates that grape- and berry-derived polyphenols exert modest but consistent improvements in CV risk markers, particularly endothelial function, with more pronounced effects observed in metabolically at-risk populations, e.g., those with MetS risk factors, hypercholesterolaemia, hyperlipidaemia, elevated blood pressure, or those who were overweight. Given the growing body of evidence over the past decade and rising rates of obesity and CV disease [123], increasing public awareness of the potential role(s) of consuming dark-fleshed grapes, blueberries, and fruits such as red-fleshed table grapes (red berry grapes) appears to represent a practical and evidence-informed adjunctive strategy for promoting CV health. More explicit integration of these into formal public health dietary guidelines may be warranted, particularly for individuals with elevated cardiometabolic risk. Finally, it should also be recognised that there is a need for larger and longer-term RCTs with whole, fresh grapes and blueberries that reflect habitual dietary intakes and patterns of consumption. Future research should also prioritise clinically meaningful endpoints, rather than relying predominantly on short-term surrogate markers.

## Figures and Tables

**Table 2:** RCT publications focusing on berry fruits/related bioactives and markers of CV health.

Study (Author, Year, Location)	Study Design	Sample Size	Bioactive Source	Bioactive Compound(s)	Intervention (Dosage/Amount)	Outcomes	Main Cardiovascular Findings	Jadad Quality
Keramatzadeh et al. (2025) [53]	8-week DB RCT	*n* = 55 with MS	Supplementation	Resveratrol	500 mg/d or placebo	Lipid profile and inflammatory markers	Resveratrol treatment significantly ↓ TNF-α (*p* < 0.001), and MDA (*p* < 0.001) vs. placebo	MQ
Cheng et al. (2024) [54], UK	5-week RCT	*n* = 28, 68–74 years	WBB extract	Not specified	111 mg, 222 mg, 444 mg, 888 mg/d, or placebo	CV effects	WBB extract at 222 mg produced acute ↓ in SBP and DBP compared with placebo	M to HQ
Tucci et al. (2024) [50], Italy	Laboratory RCT	*n* = 20, >60 years	Blueberry mousse	Anthocyanins	250 g, providing 480 mg of ACNs or a control	Vascular function	Blueberry consumption significantly ↑ RHI compared to control (mean difference + 0.42, 95% CI: 0.01–0.082, *p* < 0.05)	MQ
Woolf et al. (2023) [51]	12-week RCT	*n* = 43, 45–65 years	Blueberry	Polyphenols (metabolites)	22 g/d of freeze-dried highbush blueberry powder or placebo powder	Endothelial function	FMD/SRAUC increased by 96% from baseline with blueberry (*p* < 0.05), with no change in placebo; between-group difference favoured blueberry (*p* < 0.03)	M to HQ
Wood et al. (2023) [42], UK	12-week DB RCT	*n* = 61, 65–80 years	WBB freeze dried	Polyphenols/anthocyanins	26 g/d freeze-dried WBB (302 mg ACNs)	Vascular function	FMD ↑ and 24 h ambulatory SBP ↓ with WBB vs. placebo (0.86%, *p* < 0.001; −3.59 mmHg, *p* = 0.037)	HQ
Curtis et al. (2022) [46], UK	DB RCT	*n* = 45, 63.4 ± 7.4 years	Energy-dense drink and freeze-dried blueberries	Anthocyanins	26 g/d freeze-dried blueberries (equivalent to 1 cup/150 g fresh blueberries) or placebo	Cardiometabolic effects	Single-dose blueberry attenuated 24 h postprandial metabolic disturbances, ↓ glucose, insulin and TC, and ↑ HDL-C, HDL-P, and Apo-A1 (*p* ≤ 0.04)	HQ
Taladrid et al. (2022) [40], Spain	6-week randomised intervention	*n* = 29	Grape pomace seasoning	Polyphenols	2 g/d of GP seasoning or control (no seasoning)	Hypertension and glycaemia	BP and fasting blood glucose significantly ↓ (*p* < 0.05) after the seasoning intervention, but not for the control group	L to MQ
Wang et al. (2022) [55], UK	1-week crossover RCT	*n* = 37, 25.8 ± 6.8 years	Fresh blueberry, blueberry powder, and the control arm	Polyphenols	160 g/d of fresh whole blueberries (two adult portion sizes) or 20 g/d of freeze-dried blueberry powder (equivalent to 160 g of whole fresh blueberry), or a control capsule	CV health	Plasma NO_2_^−^ ↑ with whole blueberry (+68.66%) and powder (+4.34%) vs. baseline; ↓ in control (−9.10%)	M to HQ
Schon et al. (2021) [56], Germany	16-week DB PC RCT	*n* = 80, 40–70 years	GSE tablets	Polyphenols	300 mg/d or placebo	Blood pressure	GSE ↓ sICAM-1 and endothelin-1 secretion in HUVECs	H to MQ
Yousefi et al. (2021) [47], Canada	12-week PC RCT	*n* = 40, overweight	GSE	Flavonoids	300 mg/d or placebo, plus RCD	CV risk factors	GSE ↑ HDL-C and HDL-C/LDL-C ratio, ↓ LDL-C vs. placebo (*p* ≤ 0.04); also ↓ VAI, AIP, TC, and TG vs. baseline (*p* ≤ 0.04)	MQ
Curtis et al. (2019) [52], UK	6-month DB RCT	*n* = 115 older males	Blueberries	Anthocyanins	½ and 1 cup (75/150 g)	Cardiometabolic function	A daily intake of one cup of blueberries improved endothelial function (flow-mediated dilatation: *p* = 0.003) and systemic arterial stiffness (*p* = 0.04)	HQ
Odai et al. (2019) [41], Japan	12-week DB PC RCT	*n* = 30, 40–64 years with prehypertension	GSPE	Not specified	200 mg/d, 400 mg/d, or placebo	Vascular endothelial function	SBP ↓ 13 mmHg at 12 weeks (*p* = 0.028); FMD unchanged. In non-smokers, SBP, DBP, and arterial stiffness indices improved; Einc and PWV greater vs. placebo (*p* ≤ 0.03)	M to HQ
Bardagjy et al. (2018) [39], USA	4-week DB PC RCT (crossover)	*n* = 24, 18–55 years	Freeze-dried polyphenol-rich whole grape powder	Polyphenols	60 g/d freeze-dried polyphenol-rich whole GP or placebo and HFHC meal challenge	Endothelial function, inflammation, and PBMC gene expression	GP ↓ plasma endothelin-1 at 5 h (*p* < 0.05) and ↑ oxidative stress-related gene expression after HFHC meal	M to HQ
Martinez-Maqueda et al. (2018) [57], Spain	6-week intervention	*n* = 22, 20–65 years, two MetS risk factors	Grape pomace	Polyphenols	8 g/d of dried grape pomace or control	Insulin sensitivity and metabolic markers	Grape pomace improved fasting insulinaemia (*p* < 0.01), with no change in other cardiometabolic risk markers	L to MQ
Argani et al. (2016) [48], Iran	8-week DB PC RCT	*n* = 70, 21–64 years, mild to moderate hyperlipidaemia	RGSE	Proanthocyanidin complexes/flavonoids	200 mg/d of RGSE or placebo	Serum PON activity	↑ Apo-A1, HDL-C, and PON activity (*p* ≤ 0.001); ↓ TC, TG, and LDL-C (*p* ≤ 0.015)	MQ
Ono-Moore et al. (2016) [38], USA	Acute postprandial PC RCT	*n* = 23, 30 ± 3 years	Blueberry powder	Polyphenols	24.1 g and 48.2 g (equivalent to 204 servings fresh blueberries)	Inflammatory markers	Blueberry intake ↓ IL-1β and IL-6 in LPL-treated postprandial blood	M to HQ
Park et al. (2016) [43], USA	6-week DB PC RCT	*n* = 29	GSE	Not specified	Juice containing 300 mg/d GSE	BP and metabolic indices	Subjects with higher initial BP experienced greater BP reduction, nearly double the effect size	HQ
Johnson et al. (2015) [45], USA	8-week DB PC RCT	*n* = 48 postmenopausal women	Freeze-dried blueberry powder	Not specified	22 g freeze-dried blueberry powder	Blood pressure and arterial stiffness	↓ SBP and BP (*p* < 0.05 and *p* < 0.01, respectively) and arterial stiffness (*p* < 0.01), which may be due to increased nitric oxide production	HQ
Rahbar et al. (2015) [49], Iran	8-week RCT	*n* = 69 adults with hypercholesterolaemia	Red and white whole grapes	Not specified	500 g Condori red or Shahroodi white grapes or a control group	Oxidative markers and lipidemic parameters	Whole grapes ↓ oxidative stress (↓ TBARS, ↑ TAC); red grapes additionally ↓ total cholesterol and LDL-C, with no effect on glucose, TG, or HDL-C	LQ
Vaisman and Niv (2015) [44], Israel	12-week DB PC RCT	*n* = 50 with pre/mild hypertension	Red grape cell powder	Polyphenols	200 or 400 mg red grape cell powder	CV parameters	RGC consumption improved FMD (*p* = 0.013), reduced lipid peroxidation after 12 weeks (*p* = 0.013), and lowered diastolic BP in the 200 mg group vs. placebo (*p* = 0.032)	MQ

Key: ACNs, anthocyanins; AIP, atherogenic index of plasma; Apo-A1, apolipoprotein A1; BP, blood pressure; CV, cardiovascular; DB RCT, double-blind randomised controlled trial; DB PC RCT, double-blind placebo-controlled randomised controlled trial; DBP, diastolic blood pressure; Einc, incremental elastic modulus; FMD, flow-mediated dilation; FMD/SRAUC, flow-mediated dilation normalised to shear rate area under the curve; GP, grape powder; GSE, grape seed extract; GSPE, grape seed proanthocyanidin extract; HDL-C, high-density lipoprotein cholesterol; HDL-P, high-density lipoprotein particle; HFHC meal, high-fat high-carbohydrate meal; HUVECs, human umbilical vein endothelial cells; IL-1β, interleukin-1 beta; IL-6, interleukin-6; LDL-C, low-density lipoprotein cholesterol; LPL, lipoprotein lipase; MDA, malondialdehyde; MetS, metabolic syndrome; MS, multiple sclerosis; NO_2_^−^, nitrite; PC RCT, placebo-controlled randomised controlled trial; PBMC, peripheral blood mononuclear cell; PON, paraoxonase; PWV, pulse wave velocity; RCD, restricted calorie diet; RCT, randomised controlled trial; RGSE, red grape seed extract; RHI, reactive hyperaemia index; SBP, systolic blood pressure; sICAM-1, soluble intercellular adhesion molecule-1; SRAUC, shear rate area under the curve; TAC, total antioxidant capacity; TBARS, thiobarbituric acid reactive substances; TC, total cholesterol; TG, triglycerides; TNF-α, tumour necrosis factor alpha; VAI, visceral adiposity index; WBB, wild blueberry and ↑ increased and ↓ decreased. Jadad Quality: 0–2, low quality (LQ); 3, moderate quality (MQ); and 4–5, high quality (HQ).

## Data Availability

No new data were created or analyzed in this study. Data sharing is not applicable to this article.

## Abbreviations

The following abbreviations are used in this manuscript:

AMSTAR 2	A measurement tool to assess systematic reviews (version 2)
BP	Blood pressure
CRP	C-reactive protein
CV	Cardiovascular
CVD	Cardiovascular disease
FMD	Flow-mediated dilation
IL-6	Interleukin-6
LDL	Low-density lipoprotein
MA	Meta-analysis
PRISMA	Preferred Reporting Items for Systematic Reviews and Meta-Analyses
SR	Systematic review
sICAM-1	Soluble intercellular adhesion molecule-1
TBARS	Thiobarbituric acid reactive substances
TNF-α	Tumour necrosis factor alpha
UK	United Kingdom

## Appendix A

**Table A1:** Applied Search Words and Terminologies.

Berry fruit/bioactive	((“Grapes”[Mesh] OR grapes OR “red-fleshed grapes”) OR (“Vaccinium”[Mesh] OR blueberries OR blueberry))
	AND (“Polyphenols”[Mesh] OR “Flavonoids”[Mesh] OR flavonoids OR anthocyanins OR polyphenols)
CVD outcome	AND (“Cardiovascular Diseases”[Mesh] OR “Endothelium, Vascular”[Mesh] OR “Blood Pressure”[Mesh] OR “Platelet Aggregation”[Mesh] OR “Thrombosis”[Mesh] OR “Dyslipidemias”[Mesh] OR endothelial function OR “LDL oxidation” OR inflammation OR “lipid profile” OR “oxidative stress” OR “flow-mediated dilation” OR “arterial stiffness”)
Type of publication	AND (“Randomized Controlled Trial”[Publication Type] OR “Meta-Analysis”[Publication Type] OR “Systematic Review”[Publication Type] OR RCT)

Time restriction from 1 January 2015 to 30 April 2026.
